# Clinical Profile and Endoscopic Findings in Patients with Upper Gastrointestinal Bleed Attending a Tertiary Care Hospital: A Descriptive Cross-sectional Study

**DOI:** 10.31729/jnma.4967

**Published:** 2020-06

**Authors:** Subash Bhattarai

**Affiliations:** 1 Unit of Medical Gastroenterology, Department of Medicine, Manipal College of Medical Sciences, Pokhara, Nepal

**Keywords:** *endoscopy*, *esophageal and gastric varices*, *peptic ulcer*, *upper gastrointestinal trac*

## Abstract

**Introduction:**

Upper gastrointestinal bleeding is a common acute medical emergency. Endoscopy is the gold standard diagnostic and therapeutic tool in the management of upper gastrointestinal bleed. This study was undertaken to address the clinical profile, endoscopic profile, and outcomes in patients with upper gastrointestinal bleed.

**Methods:**

A descriptive cross-sectional study was conducted in a tertiary care teaching hospital in Gandaki Province, Nepal from January 2018 to December 2019 after obtaining ethical clearance from Institutional Review Committee (MEMG/IRC/291/GA) and informed consent from the patient or patient relatives. The sample size was calculated. Six hundred and sixty patients with upper gastrointestinal bleed were included in the study. Data entry was done in Statistical Packages for the Social Sciences version 20.

**Results:**

Peptic ulcers and ruptured oesophageal varices are the common aetiologies of upper gastrointestinal bleed. Inpatient mortality was seen in 98 (14.8 %) patients. Upper gastrointestinal bleed of variceal etiology presents with a higher Rockall score and has more chances of rebleeding and has higher mortality than those with non-variceal aetiologies. Bad prognostic factors were rebleeding, variceal etiology, and comorbidities including cirrhotic and Rockall score > 6.

**Conclusions:**

Upper gastrointestinal bleeding is a common acute medical emergency. Early upper gastrointestinal endoscopy preferably within 24 hours is recommended for diagnosis, timely intervention, and management of the patients with an upper gastrointestinal bleed that helps in reducing morbidity and mortality.

## INTRODUCTION

Upper gastrointestinal (UGI) bleed is defined as bleeding proximal to the ligament of Treitz.^1^ It is one of the common medical emergencies that have an incidence of 50 to 150 cases per 100 000 population and hospital mortality of approximately 7% to 10% even in developed countries. ^2,3^ Endoscopy is the gold standard diagnostic and therapeutic tool in the management of UGI bleed.

Non-variceal UGI bleeding is the most common cause where peptic ulcer disease accounts for 50% to 70%.^4^ Rebleeding after hospital admission is associated

with higher mortality. Rockall Scoring system can be used to predict the rebleeding and prognosis in patients with UGI bleed.^5^ A descriptive cross-sectional study regarding upper gastrointestinal bleeding is scanty from this region of Nepal.

In the present study, we will try to identify the prevalence, etiology of acute UGI bleed, clinical profiles of the patients including clinical outcomes and mortality in patients admitted in a tertiary care Hospital in Gandaki Province, Nepal.

## METHODS

This descriptive cross-sectional study was carried out in the Unit of Medical Gastroenterology, Department of Medicine at a tertiary care Teaching Hospital at Pokhara, Gandaki Province, Nepal of 24 months duration period from January 2018 to December 2019. The sample size was collected using the formula,

n = (Z^2^ × p × q)/e^2^

   = (1.96^2^ × 0.5 × 0.5)/0.05^2^

   = 384.16 ~ 385

where,

        Z = 1.96 (At 95% confidence interval)
        p = taking 50% prevalence
        q = 1-p
        e = margin of error, 5%

The calculated minimum sample size was 385. A total of 2880 endoscopies were performed in 24 months which was the sample size more than adequate for the study.

Cases were studied from the records of the endoscopy unit and departmental records of admission and discharge summaries. Patients who presented with a history suggestive of acute upper gastrointestinal bleeding i.e. haematemesis, melena, or syncope were hospitalized randomly irrespective of age, sex, or comorbidities in the emergency department, the hemodynamic assessment was done with careful measurement of pulse and blood pressure including orthostatic changes and urine output. Patients were first hemodynamically stabilized; blood transfusion was given when required. Data considering demographic variables, clinical features, bleeding characteristics were collected and alongside blood investigations like complete blood count, platelets count, blood grouping, liver function test, prothrombin time/international normalized ratio (PT/ INR), coagulation profile and ultrasonography of abdomen reports were recorded.

After hemodynamic stability, usually within 24 hours, each patient had undergone endoscopic investigation by standard flexible gastro duodenal endoscope (PENTAX EPK 700, PENTAX JAPAN Inc) and diagnostic findings were documented. The lesion with recent stigmata of hemorrhage or active bleeding was considered the cause of bleeding when various lesions were found in UGI endoscopy. For control of bleeding, pharmacologic and endoscopic treatments were used. Non-variceal bleeders were treated with continuous intravenous infusion of proton pump inhibitor for at least 72 hours and ulcer healing agents. Variceal bleeders were treated with injection terlipressin/octreotide +/-variceal band ligation. The biopsy was performed in all patients with growth, ulcers, mucosal erosions, and areas suspicious to harbor neoplasia.

Finally, the clinical presentation of patients, their endoscopic findings, complications during hospitalization including rebleeding, days of ICU stay, total hospital stay, outcomes, and mortality were assessed. All patients with acute upper GI bleed aged above 16 irrespective of sex and presentation were included in the study. The following cases were excluded from the study: 1. Patients with incomplete records 2. The same patient readmitted and endoscopy showing the same endoscopic finding in readmissions.

The study was approved and verified by the Institutional Review Committee and the work was following the rules and regulations laid down by the Institutional Review Committee. Informed consent was obtained from patients or patient relatives. Data were collected on a structured proforma covering the relevant subjects of the study and entry was done in Statistical Packages for the Social Sciences version 20. All categorical data were expressed in percent and absolute number. All numerical continuous data were expressed in mean±SD. The data analysis was done using SPSS version 20. All tests were analyzed with a 95% confidence interval.

## RESULTS

The total upper GI endoscopies performed was 2880 during the 24 months of the study period from January 2018 till December 2019. Out of these 2880 sample study, 720 patients presented with an upper GI bleed, detecting a prevalence of 25%.

But 24 patients were taken away to home or elsewhere by patient relatives against medical advice despite initial management, UGI endoscopy, and few days of admission, and 16 were excluded because of inadequate data. Twenty patients were readmitted with an upper GI bleed but had the same endoscopic findings as in previous endoscopies and admissions. Finally, a total of 660 patients; 384 (58.2%) males, and 276 (41.8%) females (M: F=1.4:1) were enrolled in the study. The mean age of subjects was 49±12.64 years with a range of 14-88 years of age. Patients were further classified as per age/sex groups with maximum cases in 40-59 years of age group (Table 1).

**Table 1:** Age groups/sex distribution of patient’s understudy.

SEX		Age groups			Total
	<40 yrs	40-59 yrs	60-79 yrs	±80 yrs	
MALE	134	195	43	12	384
FEMALE	96	137	37	6	276

These patients were classified according to their ethnicity/caste. The majority comprised of 210 (31.8%) Mongols followed by 122 (18.4%) Chhetris, 111 (16.8%) Dalits, 87 (13.2%) Newars, and 50 (7.6%), Brahmins. Other caste included 80 (12.2%) patients.

These patients were classified as per their occupation. The majority of these patients were 223 (33.8%) farmers followed by 180 (27.2%) retired personnel, 161 (24.4%) jobholders, and 96 (14.6%) housewives.

The majority comprising of 385 (58.4 %) patients were from rural areas whereas the rest 275(42.6%) belonged to urban regions. The majority comprising of 396 (60%) patients were from middle socioeconomic status. Risk factors in the study participants include smoking in 396 (60%), chronic and regular alcohol consumption in 360 (54.5 %), recent consumption (within a week) of NSAIDs and steroids in 132 (20%) and history of jaundice in 158 (24 %).

In the present study, 570 (86.4%) patients had some form of comorbid conditions (Table 2).

**Table 2:** Comorbidities of the study subjects.

Comorbidities	n (%)
Chronic liver disease liver	300 (45.5)
chronic kidney disease	20 (3)
Ischemic heart disease	64 (9.7)
History of CVA	36 (5.5)
COPD / Bronchial Asthma	18 (2.7)
Arthritis	28 (4.2)
Diabetes Mellitus	40 (6)
Hypertension	50 (7.6)
Pregnancy	14 (2.1)
No significant past medical history	90 (13.7)
Total	660 (100)

Chronic liver disease is seen in 300 (45.5 %) patients, was the most common comorbid condition identified. Chronic alcohol consumption leading to alcoholic liver disease was the most common etiology of chronic liver disease and seen in 276 (92%) patients. Chronic viral hepatitis was etiology in 16 cases (5.2%; Chronic Hep B 3.7% and chronic Hep C 1.5%). Rest 8 (2.8 %) patients were classified as cryptogenic.

Two hundred and sixty-four (40%) patients presented with only malena, 201 (30.5 %) with both haematemesis and malena, and 135(20.5 %) presented with only haematemesis. The rest 60 (9 %) had a history of dizziness, generalized weakness. Stool for occult blood was positive in these cases. A total of 126 (19%) patients presented with shock.

Among the clinical signs at presentation, pallor was the most common finding in 528 (80 %), followed by abdominal tenderness in 385 (58.36%), splenomegaly in 300 (45.4%), ascites in 240 (36.4%), clinically icterus in 280 (42.4%) and hepatomegaly in 83 (12.6 %) patients. Mean hemoglobin at presentation was 7.3 gm% (range of 3.2 gm% to 14 gm %). Haemoglobin was <10 gm% in 476 (72.16%) subjects. The mean platelet count was 96,000 / mm^3^ with a minimum of 10,000/ mm^3^ and a maximum of 5,10,000/mm^3^.

In the majority comprising of 580 (87.9%) study participants, random blood sugar levels were normal, and blood urea and creatinine were raised in almost half of the patients. Liver function tests revealed hyperbilirubinemia in 300 (45.4 % ) patients and increased ALT, AST, and prolonged prothrombin time by more than 4 sec in 350 (53%), 380 (57.6 %), and 320 (48.5%) patients respectively.

Peptic ulcer disease was the most common cause of upper GI bleed and was detected in 236 (35.7%) patients (Table 3). Peptic ulcer was seen almost equally among both the sexes with a mean age of 36±8.25 years. Duodenal ulcer was more common than gastric ulcer in both the sexes. They were further classified according to Forrest classification. Ten (4.2%) had oozing hemorrhage, 32 (13.6%) had an adherent clot, 839 (35.1%) had haematin on ulcer base and rest 111 (47.1%) had clean ulcer base and were classified as Forrest IB, IIB, IIC and III respectively. Eight out of 10 (80%) patients were injected injection adrenaline for hemostasis in oozing bleeders that stopped ongoing bleed.

Variceal bleed was the second most common cause of upper GI bleed and was detected in 224 (33.9%) (Table 3). Varices were more common in males (M: F =2.3:1) with a mean age of 49±8.34 years. One hundred and eighty-six (83%) patients were cirrhotics, predominantly alcohol-induced; 20 (8.9%) were alcoholic hepatitis, and rest 18 (8.1%) patients were classified as noncirrhotic portal hypertension. Variceal bleed was predominantly from ruptured oesophageal varices. Out of 224 variceal bleeds, 215 (96%) bled from ruptured oesophageal varices. Endoscopic Oesophageal variceal band ligation was applied in 133 (59.4%) with stigmata of ongoing bleed or varices to likely rebleed or with large varices.

Bleeding from portal hypertensive gastropathy, Mallory Weiss tear, and erosive mucosal disease accounted in 40 (6.1%), 22 (3.3%), and 62 (9.4% ) subjects respectively (Table 3). The common erosive mucosal disease was erosive gastritis followed by oesophagitis and duodenitis. No source of bleeding could be identified in 34 (5.2%) patients by upper GI endoscopy. Upper GI malignancy was seen in 42 patients and accounted for 6.4%. Upper GI malignancies were more common in males (M: F =4:3) with a mean age of 67±8.19 years. Thirty (71.4%) patients were aged between 60 -79 years of age. Five cases were aged below 40 years and they all had carcinoma of the stomach. The youngest patient was a 34 year male with gastric carcinoma. The most common upper GI malignancy over all ages and in both the sexes was carcinoma of stomach followed by oesophageal and duodenal. A gastrointestinal stromal tumor (GIST) was seen in 5 (0.7%) patients.

**Table 3:** Distribution of subjects according to etiology of UGI bleed.

Etiology of UGI bleed	Male	Female	Total	%
Gastro-oesophageal varices	156	68	224	33.9
Oesophageal Varices	150	65	215	32.6
Gastric varices	6	3	9	1.3
Peptic ulcer diseases	120	116	236	35.7
Duodenal ulcer	66	62	128	19.4
Gastric ulcer	54	54	108	16.3
Portal Hypertensive Gastropathy	24	16	40	6.1
Erosive mucosal lesions	36	26	62	9.4
Erosive gastritis	15	11	26	3.9
Erosive oesophagitis	13	11	23	3.5
Erosive duodenitis	8	5	13	2
Mallory Weiss tear	10	12	22	3.3
GI malignancies	24	18	42	6.4
Gastric carcinoma	12	10	22	3.4
Oesophageal Carcinoma	6	5	11	1.7
Duodenal Carcinoma	2	2	4	0.6
GIST	4	1	5	0.7
None identified	14	20	34	5.2
TOTAL	384	276	660	100

Among 360 alcoholics and patients with chronic liver diseases, bleeding from ruptured gastroesophageal varices were commoner than peptic ulcer bleed. Bleeding from ruptured varices, peptic ulcer, portal hypertensive gastropathy, mucosal erosive disease, MWT were seen in 153(42.5%), 139 (38.6%), 29 (8.1%), 19 (5.2%), 10 (2.8%) respectively. Among rest 10 (2.8%), no source of bleeding could be identified by UGI endoscopy.

Mean complete Rockall score after UGI endoscopy in all the subjects under study was 4.86±1.18. One Hundred and seventy-two (26%) had Rockall score more than or equal to 5.

Ninety-eight (14.8%) patients with upper GI bleed had inpatient mortality. Two third of the mortality accounting to 74 in number occurred within 7 days. Cirrhotics with complications and variceal bleeders accounted for 72 mortalities. Only 4 mortalities occurred in patients with peptic ulcer bleed. Twelve mortalities occurred in patients with GI malignancies. The rest 10 mortalities occurred in patients with pre-existing comorbidities. The most common causes of mortality were rebleeding in 29 (29.6%) within 48 hours followed by hepatic encephalopathy in 24 (24.5%) hepatorenal syndrome in 22 (22.5%) and sepsis in 14 (14.3%). Hypovolaemic and cardiogenic shock, congestive cardiac failure, spontaneous bacterial peritonitis, and aspiration pneumonia were less common causes of mortality and seen in 9 (9.1%) patients. All of the patients who died had Rockall score >6. More mortalities were observed with rebleeding, variceal bleeders, comorbidities including cirrhotics, GI malignancies, and Rockall score >6 (Table 4).

Endoscopic interventions in the form of injecting adrenaline were done in 6 peptic ulcer bleeders and endoscopic oesophageal variceal band ligation in 133 patients. No surgical intervention of any kind was necessary for this study. The median hospital stay was 6.178+2.11 days. The median ICU stay was 3.21+1.37days. Five hundred and sixty-two (85.2%) patients recovered after needful management and treatment and were discharged from the hospital. Male predominance (M: F=2.3:1) was noted in subjects with variceal bleed. Patients with the variceal bleed group presenting in shock were double than those with a non-variceal bleed group. Mean complete Rockall score after UGI endoscopy was 5.66±1.20 in a variceal group and 4.06±0.98 in the non-variceal group. Rockall score more than or equal to 5 was 3 times more in the variceal group compared to no variceal bleeders. Rebleeding within 7 days was higher and 3 times more with variceal group compared to that with a non-variceal group (30 % vs 10 %). Similarly, mortality was also higher and almost 5 times more with variceal group compared to that with a non-variceal group (31.25% vs 6.4%). (Table 5)

**Table 4:** Factors associated with mortality in patients with UGI bleed.

Parameters	Total	Discharged	Mortalit (%)
Rebleeding	29	8	21 (72.4)
Rockall score >6	140	82	58 (41.4)
Co morbid conditions	570	334	236 (41.4)
GI malignancies	36	24	12 (33.3)
Variceal bleed	224	154	70 (31.2)
Cirrhotics	300	224	76 (25.3)
Non Cirrhotics	360	338	22 (6.1)

**Table 5:** Comparison of parameters between variceal and non-variceal cirouns.

Parameters	Variceal bleed group n (%)	Non-variceal bleed group n (%)
Male: Female	2.3:1	1.1:1
Mean Haemoglobin	6.71±1.63	8.18±1.48
Platelets count <90000/mm^3^	144 (64.3)	46 (10.6)
PT prolonged by >4 sec	176 (78.6)	54 (12.4)
Shock (SBP< 90 mm Hg)	64 (28.6)	62 (14.2)
Mean Rockall score	5.66±1.20	4.06±0.98
Rockall score > 5	30 (37.5)	5 (12.5)
Rebleeding within 7 days	24 (30)	4 (10)
Death	70 (31.25)	28 (6.4)

## DISCUSSION

The mean age of subjects was 49±12.64 years with a range of 14 88 years of age with male dominance in this study. Varied age groups at presentation with UGI bleed have been mentioned in various literature. Anand et al.^6^ Rao et al.^7^ and Salih et al^8^ reported mean ages of 41, 43and 44.6 years respectively. Kaliamurthy et al.^9^ reported higher mean age of 55 years. A published UK audit showed an even higher mean age of 64.4 years.^10^ Studies reported by Gurung et al.^11^ in Dhulikhel, Nepal, and Dewan et al.^12^ in Chitwan, Nepal showed mean ages of 45.32±18.47 and 48.76±17.19 years respectively which are almost similar to the finding in the current study. The male predominance of 79% and 59% were observed in a Sudan study by Salih et al.^7^ and UK audit^10^ respectively. The male predominance of 75% and 78.4% were reported by Dewan et al.^12^ and Kashyap et al.^13^ in Nepal. The current study also showed the male predominance of 70% which was marginally less compared to the previous studies in Nepal.

Peptic ulcer disease was the most common cause of upper GI bleed and was detected in 236 (35.7%) patients in the current study. Whereas, studies by Anand et al.,^6^ Jain et al.^14^ and Rao et al.^7^ reported esophageal varices as the most common cause of UGI bleed and found that the incidence was 45.5%, 47.4%, and 51% respectively. The study by Dewan et al.^12^ in Chitwan, Nepal also revealed that esophageal varices (47.5%) were the most common cause of UGI bleed. This was followed by peptic ulcer disease (33.3%), erosive mucosal disease (11.6%), Mallory Weiss tear (4.1%) and malignancy (3.3%).^12^

The incidence of peptic ulcer bled reported by Anand et al.^6^ and Rao et al.^7^ were 38.5% and 28% respectively. Peptic ulcer bleeds accounting to 41% was the common cause reported by Kaliamurthy et al.^9^ from Jamaica. The incidence of peptic ulcer bleed was 36% in a UK audit ^10^ and 30.6% by Bhutta et al.^15^ Peptic ulcer disease detected in 35.7% was the most common cause of upper GI bleed followed by 33.9%of variceal bleeds in the current study.

Bleeding from erosive mucosal disease was 9.4% in the current study. Anand et al.^6^ and Rao et al.^7^ found the erosive mucosal disease in 8.5% and 9% respectively. The incidence rates were almost similar in the above-mentioned studies. But Bhutta et al.^15^ found a higher incidence rate of 18.4%. Bleeding from Mallory Weiss tear was found to be 3.3% in the current study. Kashyap et al.^13^ reported Mallory Weiss to tear as a cause of upper GI bleeding in 12 patients (10.8%) and Bhutta et al.^15^ reported it in 2 patients (1.4%).

Bleeding from upper GI malignancy was 6.4% in the current study. It was reported to be 5.9 % and 3.3 % in the studies by Jain et al.^14^ in India and Dewan et al.^12^ in Chitwan, Nepal respectively. No etiology of upper GI bleed was identified in 5.2% in the current study. Whereas, 16.1% had normal endoscopic findings in an Indian study by Jain et al.^14^

Mean hemoglobin at presentation was 7.3 gm% in the current study. It was 8.4 gm% in the study by Jain et al.^14^

Among 380 chronic alcohol consumers and patients with chronic liver diseases, bleeding from ruptured gastroesophageal varices was the most common cause of bleed followed by peptic ulcer bleed in our current study. Bleeding from ruptured varices, peptic ulcer, portal hypertensive gastropathy, mucosal erosive disease, MWT were seen in 153 (42.5%), 139 (38.6%), 29 (8.1%), 19 (5.2%), 10 (2.8%) respectively. In a similar study in central Nepal by Bhattarai et al.,^16^ ruptured oesophageal varices (66.7%) was the most common cause of UGI bleeding in patients with chronic liver disease followed by peptic ulcer bleeding in 19.2%.

In the current study, 40% of patients presented with only malena, 30.5% with both haematemesis and malena, and 20.5% presented with the only haematemesis. The rest 9% had a history of dizziness and generalized weakness. A total of 19% of patients presented with shock in the current study. In the study by Dewan et al.,^12^ both haematemesis and malena was seen in 71.7%, only malena in 20% and only haematemesis in 8.3% at presentation. Shock was detected in 21.7% of patients with UGI bleed in that study.

Among patients with chronic liver disease and variceal bleed, this subset of patients presented with severe anemia, shock, and more complications. Rockall score was higher, rebleeding rates were more (30 % vs 10 %) and higher mortality almost 5-fold (31.25% vs 6.4%) was recorded in variceal bleeders on comparison with non-variceal bleed in the current study. Similar were the findings in the study done by Bhattarai et al.^16^ in central Nepal with increased mortality amongst variceal bleeders (27.5 % vs. 7.5%). Similarly, mortality was higher in variceal bleed group (20.2% vs 6.9 %) in the study by Svoboda et al.^17^

In the current study, the overall mortality was seen in 98 (14.8 %) patients. Salih et al.^8^ investigated 238 patients and found a mortality rate of 3.4%. Kashyap et al.^13^ studied 111 patients in India and showed an overall mortality rate of 3.6% and all patients had esophageal variceal bleed. Kaliamurthy et al.^9^ reported a mortality rate of 5.7%, whereas UK Audit 2007^10^ reported a mortality of 10%. Mortality of 4.2% was reported in the study by Dewan et al. in Chitwan, Nepal. The mortality rate was higher in the current study when compared to the above mentioned studies.

The median hospital stay was 6.178±2.11 days in the current study whereas, it was 7.28±3.18 days in the study by Dewan et al.^12^ Rebleeeding, presence of comorbidities and Rockall score >6 were the identifiable risk factors for mortality in the study by Dewan et al.^12^ These risk factors were also associated with mortality in our study patients. Advanced age >60 years, variceal bleeders, deranged coagulation profile, prolonged prothrombin time, thrombocytopenia were the other factors associated with mortality in the current study.

This study had its limitations. The study reflects a certain geographical area. Most of the patients had no repeat endoscopies records on follow-ups. Etiology of UGI bleed could not be identified in some subjects because of the unavailability of enteroscopy, capsule endoscopy, and other needful modalities. Early and appropriate fluid and pharmacological management alongside upper GI endoscopy preferably within 24 hours are recommended for diagnosis, timely intervention, and management of the patients with UGI bleed that helps in reducing morbidity and mortality. More expertise with training and availability of diagnostic and therapeutic endoscopies in the majority of hospitals will help in reducing complications and mortality secondary to UGI bleed. This study may help formulate guidelines for proper and needful management of upper gastrointestinal bleed.

## CONCLUSIONS

Peptic ulcers and ruptured oesophageal varices were common aetiologies of upper GI bleed. Inpatient mortality was seen in 98 (14.8 %) patients. UGI bleed of variceal etiology presented with a higher Rockall score and had more rebleeding and had higher mortality than those with non-variceal aetiologies. Bad prognostic factors were advanced age >60 years, massive rebleeding, variceal bleeders, deranged coagulation profile, prolonged prothrombin time, thrombocytopenia, comorbidities including cirrhotics and Rockall score >6. Furthermore, the Rockall score is an effective tool for outcome prediction in patients with acute upper GI bleeding.

## Conflict of Interest:

**None.**
